# The reliability and prognostic implications of a simplified bone age classification system for adolescent idiopathic scoliosis

**DOI:** 10.1186/1748-7161-7-S1-O14

**Published:** 2012-01-27

**Authors:** L Dolan, K Masrouha, G El-Khoury, S Weinstein

**Affiliations:** 1 University of Iowa, Department of Orthopaedic Surgery, Iowa City, IA 52242-1007, USA; 2 University of Iowa, Department of Radiology, Iowa City, USA

## Background

Sanders et al. [1,2] describe a simplified system for determining digital skeletal age (DSA) and its use in predicting the likelihood a curve will reach surgical magnitude. We assessed the inter-and intra-rater reliability and prognostic implications of this classification system using data from a multicenter trial of outcomes in AIS (BrAIST).

## Material and methods

36 subjects were randomly selected. We determined the predicted prognosis by cross-classifying the DSA and Cobb angle using Sanders’ estimates.

## Results

Kappa coefficients ranged from 0.76 to 0.88. For example, one rater’s reading corresponded to a 0% risk of the curve reaching surgical magnitude, while the other rater’s reading for the same subject corresponded to a 92% risk.

The high level of agreement in DSA found by Sanders et. al was replicated in this study, and would be considered “substantial” to “almost perfect” using widely applied standards [3] Despite this agreement, different prognoses were predicted for 11% of these subjects.

## Conclusions

Clinicians and researchers should consider seeking a second review of the DSA (especially if it appears to be in the DSA 2 to 3 range), and the Cobb angle, prior to using it to make prognostic predictions and treatment decisions.
